# Genome sequencing and analysis of Mangalica, a fatty local pig of Hungary

**DOI:** 10.1186/1471-2164-15-761

**Published:** 2014-09-05

**Authors:** János Molnár, Tibor Nagy, Viktor Stéger, Gábor Tóth, Ferenc Marincs, Endre Barta

**Affiliations:** Agricultural Genomics and Bioinformatics Group, Agricultural Biotechnology Institute, NARIC, Gödöllő, Hungary; Biomi Ltd, Gödöllő, Hungary; Institute of Enzymology, Hungarian Academy of Sciences, Budapest, Hungary; Hungarian Scientific Research Fund, Budapest, Hungary

**Keywords:** Mangalica, Genome sequencing, Fatty pig, Breed-specific SNP, Gene function

## Abstract

**Background:**

Mangalicas are fatty type local/rare pig breeds with an increasing presence in the niche pork market in Hungary and in other countries. To explore their genetic resources, we have analysed data from next-generation sequencing of an individual male from each of three Mangalica breeds along with a local male Duroc pig. Structural variations, such as SNPs, INDELs and CNVs, were identified and particular genes with SNP variations were analysed with special emphasis on functions related to fat metabolism in pigs.

**Results:**

More than 60 Gb of sequence data were generated for each of the sequenced individuals, resulting in 11× to 19× autosomal median coverage. After stringent filtering, around six million SNPs, of which approximately 10% are novel compared to the dbSNP138 database, were identified in each animal. Several hundred thousands of INDELs and about 1,000 CNV gains were also identified. The functional annotation of genes with exonic, non-synonymous SNPs, which are common in all three Mangalicas but are absent in either the reference genome or the sequenced Duroc of this study, highlighted 52 genes in lipid metabolism processes. Further analysis revealed that 41 of these genes are associated with lipid metabolic or regulatory pathways, 49 are in fat-metabolism and fatness-phenotype QTLs and, with the exception of *ACACA*, *ANKRD23*, *GM2A*, *KIT, MOGAT2, MTTP, FASN, SGMS1, SLC27A6* and *RETSAT*, have not previously been associated with fat-related phenotypes.

**Conclusions:**

Genome analysis of Mangalica breeds revealed that local/rare breeds could be a rich source of sequence variations not present in cosmopolitan/industrial breeds. The identified Mangalica variations may, therefore, be a very useful resource for future studies of agronomically important traits in pigs.

**Electronic supplementary material:**

The online version of this article (doi:10.1186/1471-2164-15-761) contains supplementary material, which is available to authorized users.

## Background

Due to the economic value of farm animals, their genomics, in general, and whole genome sequencing, in particular, are important issues. Results of such research have already had an impact and will continue to do so in the future in terms of production of meat, milk, fibre and other products, environmental effects of animal husbandry, breeding, animal health, feeding, and even human medical issues such as xenotransplantation and disease modelling [1, 2]. Regarding this, the genome of a number of agriculturally important animal species has been or is being completed [3–11].

Pig is one of the most important farm animals, providing about 103,000 thousand tonnes of pork for meat consumption worldwide in 2012 [12]. Moreover, pigs can be used as a model for human diseases, such as arthritis, cardiovascular diseases, diabetes and obesity, because pigs are more similar to humans at physiological and gene level, when compared with rodent animal models [2]. According to different sources, the predicted number of pig breeds and lines range from 350 to 730 [13, 14]. Most of these breeds are local, with only 25 found in multiple regions of a country, and a further 33 spread to more than one country [13]. In spite of the larger number of pig breeds, only six (Large White, Duroc, Landrace, Hampshire, Berkshire and Pietrain) dominate the pork industry [13].

In the last decade, enormous efforts have been made to exploit the genetic and genomic resources of pigs. Genome sequencing of swine goes back to the early 2000’s, when the Sino-Danish Pig Genome Project was initiated and subsequently a 0.66× coverage genome survey, based on shotgun sequencing, was published [15]. Deeper coverage sequencing of the pig genome was initiated by the Swine Genome Sequencing Consortium [16]. The Sscrofa9 genome assembly was released in 2009 [17] and the pig genome sequence was recently published [9]. These genome resources for pig, together with specialised sequencing projects such as parallel sequencing, have had a huge impact on widening our knowledge about the pig genome, to include SNP identification and genotyping [18–20], GC variance [21], muscle transcriptome [22, 23], pig interactome [24], domestication/selection [25], evolution/domestication [9], and in a number of other recently published research topics [26].

Despite the large number of local pig breeds, only a few of them (for example Angler Satleschwein, British Saddleback, Cinta Senese, Manchado de Jabugo, Basque and Guodyerbas), were included in genome sequencing projects. In addition to the major industrial and the few local breeds, Asian and European wild boars, several Asian pig breeds and several other species of the *Sus* genus have also been included [9, 27–29]. However, other local breeds, of which many are endangered, should also be of great interest for genomic studies because of their importance in biodiversity, conservation, local community and even pork production issues [14, 30]. Mangalica is an example of a local/rare breed with a characteristic curly hair phenotype, which is indigenous to Hungary and was developed in the 19^th^ century [14]. Mangalicas are fatty-type pigs [31], with high intramuscular fat content [32]. Mangalicas have three colour variants, Blond, Red and Swallow-belly, which are considered as separate breeds based on microsatellite studies [33]. As the history of the three Mangalica breeds indicate [14], the Blond was bred first from old Hungarian pig races and pigs of Mediterranean origin, and then it contributed to the two newer breeds, Red and Swallow-belly Mangalicas. Reproduction studies are quite numerous in Mangalica [34–38], but genetic studies are rare [39]. Previously we have described that the mtDNA D-loop sequences of Mangalicas display low diversity, but the maternal lineages that they represent are genetically distant from cosmopolitan breeds kept in Hungary [14] and very likely originate from one particular European ancient line [40].

In order to explore how the genomes of Mangalicas differ from the reference pig genome, we have sequenced a male individual of each of the three Mangalica breeds along with a male Duroc individual of Hungarian origin. The genome sequence of Mangalicas can serve as a basis for future conservation of the breeds and for an extended Mangalica pork industry.

## Results

### Genome sequencing

Three Mangalica male pigs with a Mangalica-specific mitochondrial D-loop haplotype were selected [40] for genome sequencing. These animals were kept at Emőd, Hungary, registered at the Hungarian Mangalica gene-bank as pedigree sires. They were previously assessed as Blond, Red and Swallow-belly Mangalicas, respectively, under the Hungarian Mangalica Standard and by microsatellite analysis. A Duroc male of Hungarian origin was also sequenced, because we have found previously that Duroc pigs of international or Hungarian origin belong to different maternal lineages [40] and Mangalica × Duroc F1 hybrids are processed at industrial scale in Hungary for pork products.

**Figure 1:**
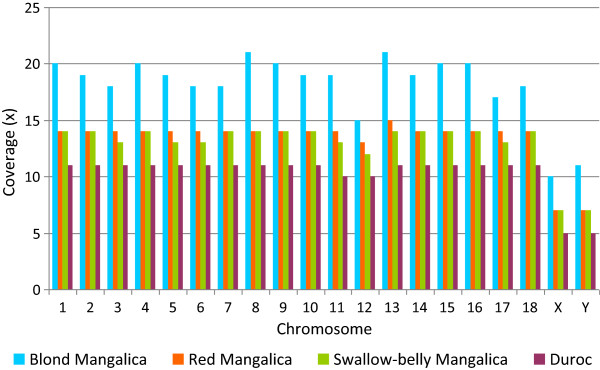
**Sequence coverage of the auto- and sex-chromosomes in four pig individuals.**

Genome sequencing resulted in 6.27 × 10^8^, 4.15 × 10^8^, 4.06 × 10^8^ and 3.32 × 10^8^ reads for the genomes of the Blond, Red and Swallow-belly Mangalica and the Duroc animals, respectively (Table 1). Due to the 500 bp average fragment size of the libraries used for the 2 × 100 bp paired-end sequencing, 300 bp long spacer between the reads was predicted. Mapping of the reads to the reference pig genome Sscrofa 10.2 resulted in an excellent correspondence between the expected and observed length of the spacers (Additional file 1). The proportion of the mapped reads was 77.3, 83.3, 82.8 and 82.5% resulting in 19×, 14×, 14× and 11× median autosomal coverage, respectively, for the four sequenced individuals (Table 1). The coverage for the individual autosomes varied between 10× and 21×, while for the sex chromosomes about half of the autosomal coverage was obtained (Figure 1). In addition, large numbers of reads for the Blond (260,270), Red (98,832) and Swallow-belly (104,478) Mangalicas and the Duroc (100,663) individual resulted in 1,571×, 602×, 638× and 615× coverage of the pig reference mitochondrial genome [41], respectively.

**Table 1:** **Sequencing statistics**

	BM ^a^	RM ^a^	SM ^a^	D ^a^
Total reads	626951708	414579434	405954574	331599252
Mapped reads	484893153	345426413	335911424	273390375
Mapped reads (%)	77.3	83.3	82.8	82.5
Autosomal median coverage	19	14	14	11

^a^BM, Blond Mangalica; RM, Red Mangalica; SM, Swallow-belly Mangalica, D, Duroc.

### Identification of genetic variants

To identify SNP and INDEL variants we used the SAMtools and GATK pipelines. In each animal, SAMtools and GATK provided a very similar number of SNPs and the proportion of the concordant variations was high. In contrast, GATK detected more INDELs than SAMtools, and thus the proportion of the common INDELs was lower compared either to the SNPs or to the total numbers of INDELs identified by the pipelines (Additional file 2). We analysed only the concordant variants further. More than seven million SNP and INDEL variants were identified by comparing the genome of each Mangalica individual to the Sscrofa 10.2 genome assembly. The genome sequence of the Duroc male also contained almost 6.5 million SNPs and INDELs when compared with the reference genome, which was assembled predominantly from a Duroc female animal [20]. SNPs outnumbered INDEL variations in all four animals by about 10-fold. In the Blond Mangalica, more homozygous then heterozygous SNPs were identified; in the Red Mangalica their number was about the same, while in the Swallow-belly Mangalica there were more heterozygous than homozygous SNPs. In the Duroc animal, there were more heterozygous than homozygous SNPs. In each individual, more homozygous than heterozygous INDELs were found and their ratio was also about the same. SNP transitions were more numerous than transversions in all four individuals by about 2-fold. A summary of the statistics for these data are shown in Table 2.

**Table 2:** **Categories of sequence variations**

	BM ^a^	RM ^a^	SM ^a^	D ^a^
SNPs	6944767	6871283	6734038	5950027
INDELs	696029	623282	617600	451299
Total variants	7640796	7494565	7351638	6401326
Heterozygous SNPs	3196568	3443594	3722921	3314982
Homozygous SNPs	3744651	3424501	3008107	2633108
SNP transitions	4782645	4739843	4641040	4097573
SNP transversions	2165670	2134628	2096008	1854391
Multiple SNPs	3548	3188	3010	1937
Heterozygous INDELs	210860	200062	167240	144505
Homozygous INDELs	464812	406643	436692	298970

^a^BM, Blond Mangalica; RM, Red Mangalica; SM, Swallow-belly Mangalica, D, Duroc.

**Figure 2:**
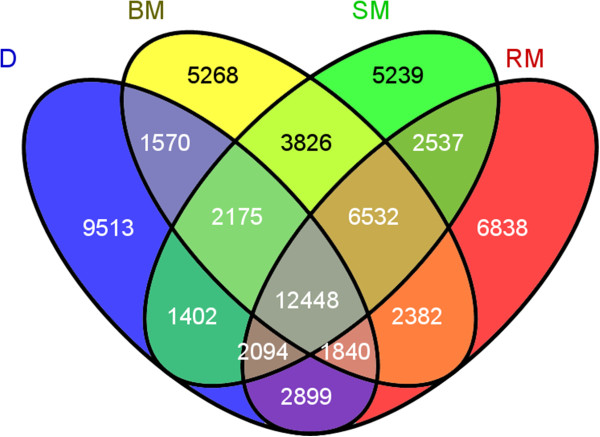
**Venn diagram of exonic SNPs in the sequenced animals.** D, Duroc; BM, Blond Mangalica; SM, Swallow-belly Mangalica; RM, Red Mangalica.

Filtering the SNP variations using stringent criteria (see Methods) resulted in 6.2 × 10^6^, 6.3 × 10^6^, 6.2 × 10^6^ and 5.4 × 10^6^ SNPs in the Blond, Red and Swallow-belly Mangalica and the Duroc individuals, respectively (Additional file 3). Approximately 9 to 13% of the filtered SNPs were revealed as novel (Additional file 3) when compared with the 28.6 million SNPs in the pig dbSNP138 database. The filtered SNPs were grouped into main and sub-categories according to their intergenic or genic position and synonymous or non-synonymous nature (Additional file 4). It was observed that Mangalicas, in contrast to the Duroc animal, had more homozygous than heterozygous variations in almost all SNP categories. A comparison of both synonymous and non-synonymous exonic SNP variants revealed 12,448 SNPs that were common to the four animals, and approximately 5,200 to 9,500 unique SNPs for each individual (Figure 2).

The detection of large INDELs was not the scope of the current study, and so only INDELs shorter than 52 bp were identified. For the genomes of the Blond, Red, Swallow-belly Mangalicas and the Duroc pig, approximately 6.9 × 10^5^, 6.2 × 10^5^, 6.1 × 10^5^ and 4.5 × 10^5^ such INDELs were identified, respectively. Of these, 99.9% were novel compared to the dbSNP138 database. With respect to the size distribution, of the INDELs among the four genomes, single base-pair INDELs were the most abundant (Additional file 5). Exonic INDELs were sorted into eight categories: frame-shift deletions, frame-shift insertions, frame-shift block substitutions, non-frame-shift deletions, non-frame-shift insertions, non-frame-shift block substitutions, stop-gains and stop-losses (Additional file 6). In exonic INDELs, apart from the relatively large number of one base-pair variations that cause ORF shifts, +/− 3 base-pair changes, which do not effect the ORF, were identified in higher numbers than two or four base-pair variations (Additional file 7). An elevated number of one base-pair INDELs when compared with other sizes has also been reported by others [42, 43]. Our comparison with the platinum human exonic data obtained from Illumina’s BaseSpace (https://basespace.illumina.com/datacentral) provided the same result (data not shown) suggesting that our analysis with the pig genome is reliable.

**Figure 3:**
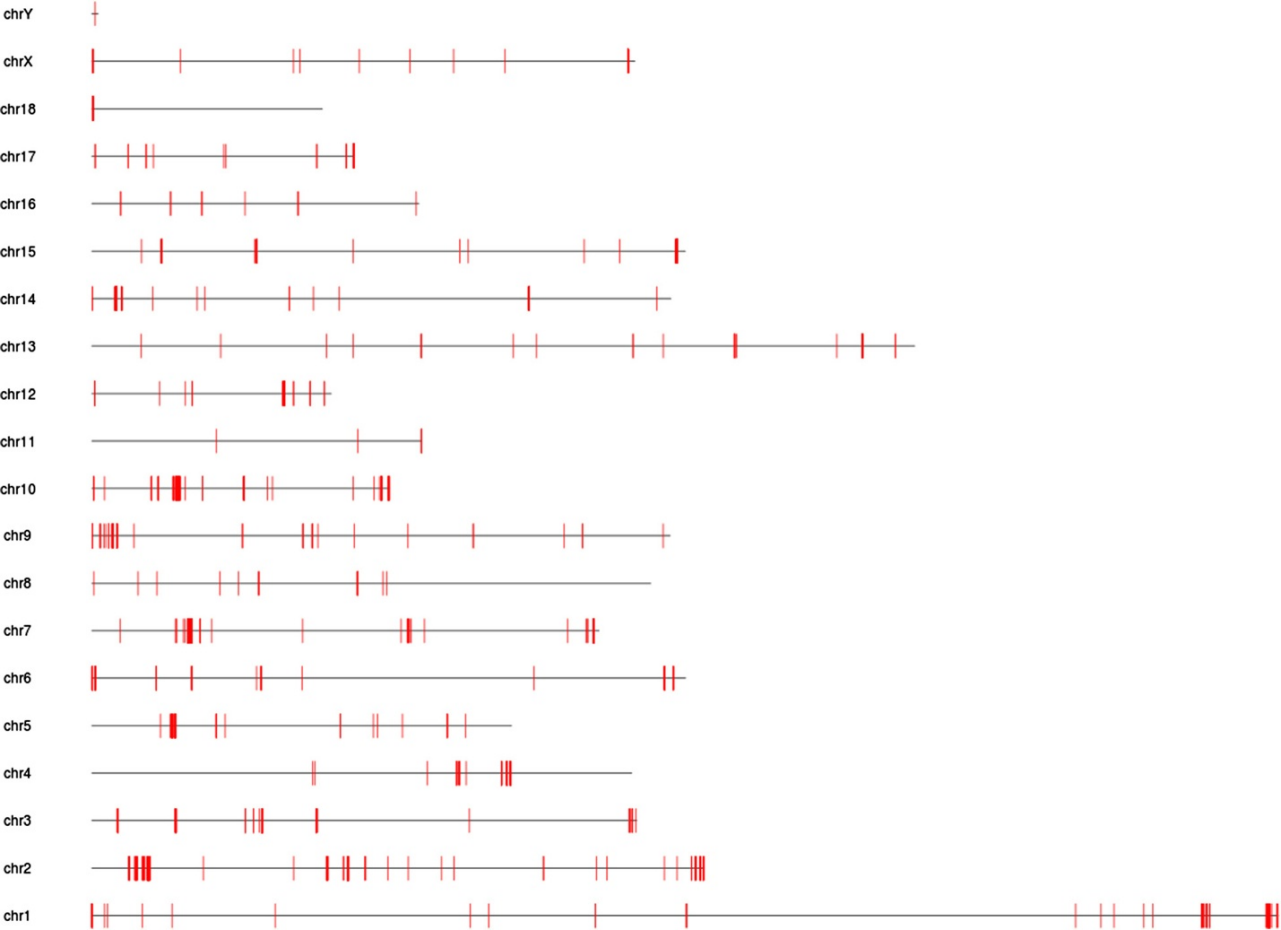
**Distribution of CNVs across Mangalica chromosomes.** Short vertical lines represent the position of CNVs, which are present in all three sequenced Mangalicas.

Copy number variants (CNVs) were identified that were common amongst the three sequenced Mangalicas. Only CNV gains were analysed further due to the effect of sequence coverage depth on CNV losses [44]. One thousand and forty-one CNV gains with a copy number of three or more were identified across all chromosomes (Figure 3). The minimum and maximum size of the CNVs was 1,000 and 135,735 bp, respectively with an average of 3,529 bp. Of the 1,041 Mangalica CNVs, 485 and 160 had no positional overlap with either the 3,118 CNV gains described by Paudel and colleagues [44] or the 145,857 CNVs identified in the Duroc animal in this study, respectively, while the numbers of overlapping CNVs were 556 and 881, respectively. We note here that the very large number of CNVs in the Duroc animal is because no statistical test could be performed on data from one individual. Porcine genes could be annotated to 155 CNVs, while 886 CNVs did not contain any gene (Additional file 8). Of the 155 genes, 150 were unique since five genes contained two CNVs. An overrepresentation analysis identified 16 out of the 150 unique genes, which were in the overrepresented Molecular function (GO:0003674) category (P value = 1.25 × 10^−7^). One of the 16 genes, *HOXB8*, encoding a homeobox protein, is neither present in the literature [44] nor in the sequenced Duroc animal used in this study (Additional file 8).

### Analysis of genes with exonic, non-synonymous SNPs

#### Functional, QTL and pathway annotation of the genes

**Figure 4:**
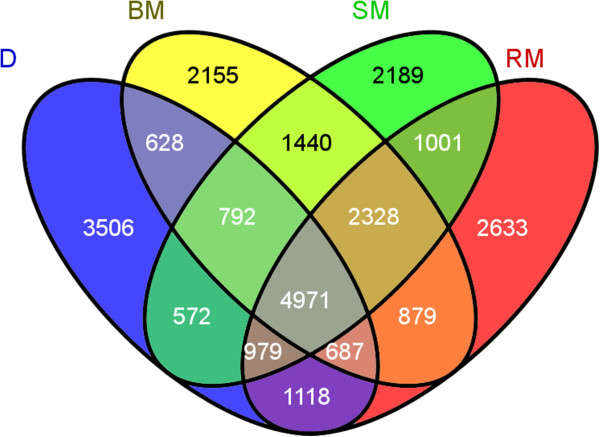
**Venn diagram of exonic, non-synonymous SNPs in the sequenced animals.** D, Duroc; BM, Blond Mangalica; SM, Swallow-belly Mangalica; RM, Red Mangalica.

**Figure 5:**
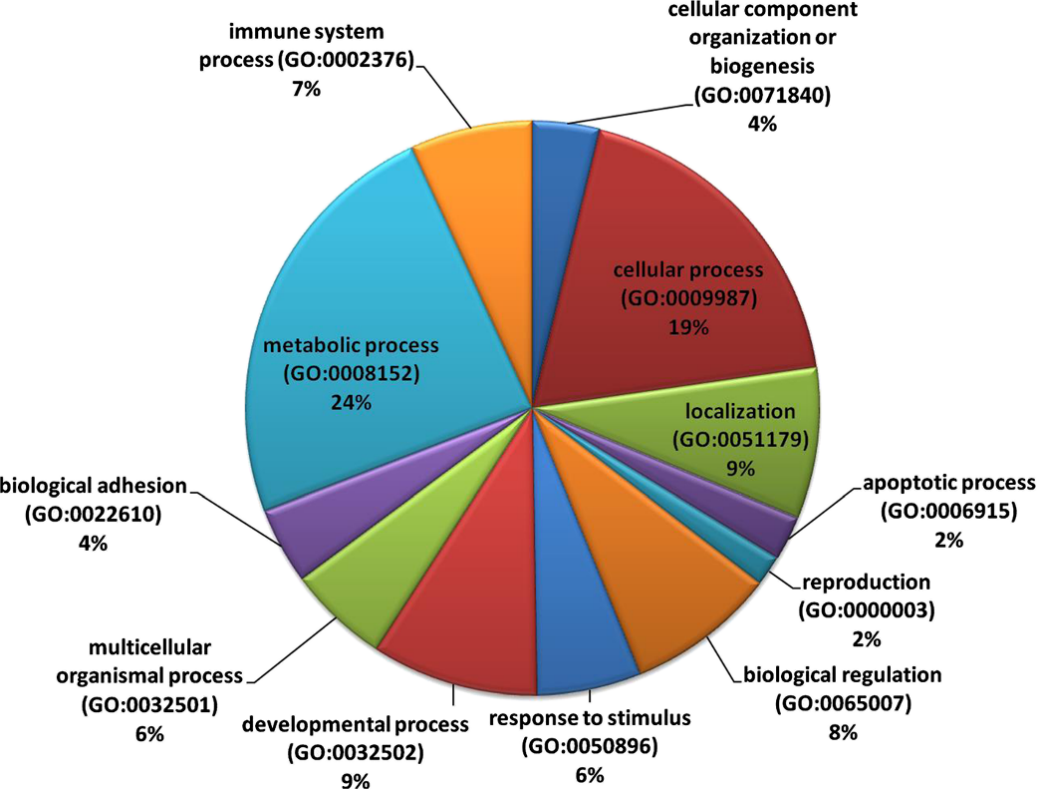
**Biological process ontology of genes with exonic SNPs found in Mangalica breeds.** Of the 1.389 genes, 1,372 resulted in 2,130 total hits in processes. Percentage indicates the percent of genes in one process against the total number of process hits.

Due to the importance of the Mangalica × Duroc hybrids to the Hungarian pork industry, the 2,328 exonic, non-synonymous SNPs common to all three Mangalica breeds but absent from sequenced Duroc animal (Figure 4) and the reference pig genome, were selected for functional analysis. These SNPs in the coding regions of genes, which result in amino acid changes in proteins, may be of great importance as they could be the polymorphisms affecting variation in phenotypes. The 2,328 SNPs were mapped to 1,389 unique genes of the Sscrofa10.2 assembly as certain genes had multiple SNPs (Additional file 9) and their annotation into biological process (BP) categories by the web-based software PANTHER [45] revealed that they belong to twelve major GO groups (Figure 5). Since the SNPs were identified by comparing Mangalicas, which are fatty-type of pigs, and Duroc, which is a lean-type breed, we were particularly interested in those SNP-harbouring genes that might be involved in fat-related biological processes. Amongst the 1,389 unique genes with exonic, non-synonymous SNPs, we have identified 52 genes, which belonged to Lipid metabolic process (GO:0006629). Although this category, in contrast to when two sets of 1,389 randomly chosen genes were used as control, appeared in an overrepresentation analysis, it was not overrepresented using the strict Bonferroni correction (Additional file 10). As another control, we have found no overrepresentation using the full pig gene set. Despite the lack of overrepresentation, we still consider that the identified genes might have a great importance, since the amino acid changes caused by the SNPs in them may affect the structure and, consequently, the function of the encoded protein, and such functional alterations of proteins remain hidden in gene expression studies. The importance of our SNP-based gene identification approach is indicated by, for example, that proteins encoded by the *PNLIP* and *PNLIPRP2* genes, which were not associated to fatness phenotypes in pigs before, are the target of Orlistat (tetrahydrolipstatin), a drug used for treating obesity in humans (data not shown). The possible effect of exonic SNPs on protein function is discussed below using FASN as an example.

To study the possible relationship between the 52 genes in the lipid metabolic process GO category and QTLs, the chromosomal position of each genes was compared to the positions of the “Fatness” and “Fat composition” QTLs downloaded from the QTLdb, Release 19, [46]. Forty-nine genes are in one or more fat-related QTLs with 14 genes on chromosome 14, overlapped by 15 fat-associated QTLs (Additional file 11). Because of this large proportion (~28%) of genes on chromosome 14, we performed an enrichment analysis for the 14-gene set and a control set of 1282 genes, both are in the same region of chromosome 14 determined by the 15 QTLs. The corrected P-value for lipid metabolic genes in the control and in our set was 4.80 × 10^−3^ and 2.95 × 10^−19^, respectively, indicating that the enrichment of the 14 genes in these QTLs deviate significantly from random.

Fatty acid composition of meats is an important dietetic and health issue for pork consumers. We, therefore, compared those genes, which are in saturated and unsaturated fatty acid QTLs and found that nine genes were in common across both fatty acid categories, while the saturated and unsaturated QTL groups each contained two unique genes, *NKX2-3* and *EPHX2*, and *OMA1* and *FAM135B*, respectively (Additional file 12).

**Figure 6:**
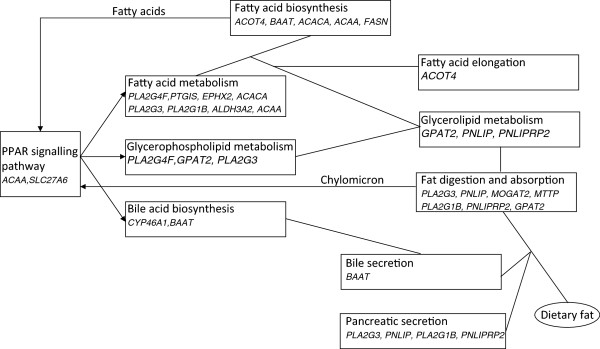
**Fat metabolic pathways and participating genes with Mangalica-specific exonic, non-synonymous SNPs.** Lines represent the interconnections of the pathways. Arrows indicate where signalling or metabolites (name above the line) affect genes in other pathways.

Of the 52 lipid metabolic process-associated genes, we could map 41 to one or more pathways using the KEGG database. Almost 44% (18) of the mapped genes were associated with lipid metabolic pathways (Figure 6), while others contribute to glycan and carbohydrate metabolisms, biochemical processes at the interface of lipid and other metabolic pathways and the regulation of lipid metabolism (Additional file 11). Of the 41 mapped genes, two are particularly important. One is *FASN*, which encodes an enzyme involved in a number of steps in the synthesis of 8 to 16 carbon-chain fatty acids in the fatty acids biosynthesis pathway [KEGG:ssc00061]. The FASN protein is a homodimeric multifunctional enzyme with six catalytic domains, which processes different steps of cyclic elongation of fatty acids [47]. The other gene is *SLC27A6*, a member of a gene family, which is expressed in liver, heart and subcutaneous backfat of pig [48]. The encoded protein is a fatty acid transporter, which is one of the two membrane proteins of the PPAR signalling cascade [KEGG:hsa03320], which regulate lipid and fatty acid metabolism, bile acid biosynthesis and adipocyte differentiation, amongst other regulated processes [49].

#### Genotyping SNPs in other breeds

The 90 SNPs in the above described 52 genes were present in all three sequenced Mangalicas, but absent from the sequenced Duroc and the reference genome. To learn about their wider occurrence, we have “e-genotyped” 55 animals whose genome was sequenced [9] for these SNPs. The results indicate that the frequencies of these SNPs vary amongst the 55 individuals (Additional file 13). Clustering of the average frequencies revealed four clusters among the individuals, where Mangalica represents a separate cluster and European, international/Hungarian Duroc, and non-European pigs and/or wild boars comprise the three other related groups (Additional file 14). The clear separation of Mangalicas from other breeds by those 90 SNPs might have the potential in practical applications, such as whole genome selection in breeding.

It was found that four SNPs are present only in Mangalicas, but not in the genotyped individuals (Additonal file 13). All of these SNPs are in one gene, *MOGAT2* (ENSSSCG00000014861), which encodes a monoacylglycerol O-acyltransferase 2 enzyme, and is in several back- and belly-fat QTLs and in the “Fat digestion and absorption” (KEGG: 04975) pathway (Additional file 11). It is possible, therefore, that this gene has a particular role of the development of the fatty-pig phenotype of Mangalicas.

Some studies have highlighted the importance of the FASN gene in pig fatness [50, 51]. In this gene, we have identified two non-synonymous SNPs, which are present in the three sequenced Mangalicas, but not in the reference genome and the sequenced Duroc individual used in this project. They are also different from those three SNPs that have been genotyped previously [50]. SNP1 is in exon 9 (chromosome 12, position 1,028,766) and is a G•C (reference) to A•T (Mangalica) transition, which causes a R443Q amino acid change while SNP2 is a C•G (reference) to T•A (Mangalica) transition in exon21 (chromosome 12, position 1,025,096) resulting in a T1088I change in the FASN protein. The frequency of these two SNPs is quite diverse in the genome sequenced animals, including the three Mangalicas and one Duroc individual sequenced in this study (Additional file 15). We, therefore, genotyped 72 Mangalica and 21 Duroc pigs for both SNPs in order to get more information about these SNPs in the two breeds. We found that the A (“Mangalica”) alleles (SNP1_A•T_ or SNP2_T•A_) occurs at a much higher frequency than the B alleles in Mangalica, whereas in contrast the B alleles (SNP1_G•C_ or SNP2_C•G_, “non-Mangalica”) are more prevalent in Duroc (Table 3). Additionally, we found that for SNP1, 62 and 10 Mangalicas and 1 and 20 Duroc animals were AA and BB homozygous, respectively; no heterozygotes were found. For SNP2, 65 Mangalicas and eight Durocs had AA, five Durocs had BB, and seven Mangalicas and eight Durocs had AB genotypes respectively; no Mangalica with BB genotype was found.

**Table 3:** **Genotyping the** ***FASN*** **gene**

	Breed	Allele (Nucleotide)	Allele frequency
SNP1	Mangalica	A (A•T)	0.86
	Mangalica	B (G•C)	0.14
	Duroc	A (A•T)	0.05
	Duroc	B (G•C)	0.95
SNP2	Mangalica	A (T•A)	0.95
	Mangalica	B (C•G)	0.05
	Duroc	A (T•A)	0.05
	Duroc	B (C•G)	0.95

## Discussion

The genome of one individual each of the three Mangalica breeds (Blond, Red and Swallow-belly), and a Duroc animal from a Hungarian herd was sequenced and analysed. More than 100 million reads were obtained from the genome of each animal. On average for the four genomes sequenced, 81% of the reads were mapped to the reference genome, resulting in 14.5× median autosomal coverage. Millions of SNP and hundred-thousands of INDEL variations were identified in the three Mangalicas and the one Duroc genome, respectively, when compared to the reference pig genome assembly Sscrofa 10.2. By filtering the SNPs, about five to six million variations were obtained, and about one-tenth of these were novel SNPs compared to the dbSNP138 database (Additional file 3).

For functional analysis, we selected 2,328 exonic non-synonymous SNPs present in each sequenced Mangalica individual, but absent from either the reference genome or the Hungarian Duroc animal. These SNPs were mapped to 1,389 pig genes present in the Ensembl database. Since Mangalicas are fatty-type pigs, and the SNPs were identified in comparison with Duroc, a lean-type pig, we were particularly interested in fat-related genes in this set. Fifty-two genes were found belonging to lipid-related metabolic process categories and were further analysed using QTL and pathway data-mining. Of the 52 genes, 49 and 41 are associated with fat-related QTL regions and KEGG pathways, respectively (Additional file 11).

Some of the 52 genes, for example *ACACA*, *ANKRD23*, *GM2A*, *KIT, MOGAT2, MTTP, FASN, SGMS1, SLC27A6* and *RETSAT*, which we have highlighted here, have been previously described in the context of fat-related characteristics in pigs [50–54]. Of these genes, *FASN*, a gene encoding a fatty acid synthase, has been shown to be associated with a *cis*-11-Eicosenoic acid (C20:1) percentage QTL in a Guadyerbas × Landrace cross, although none of the identified SNPs had any putative effect on the protein structure [50]. The FASN protein is a homodimeric, multifunctional enzyme with six catalytic domains, which are required for the cyclic elongation of fatty acids [47] and catalyses 32 reactions in the fatty acid biosynthesis [KEGG:ssc00061] pathway. Targeted mutagenesis of the *FASN* gene and inhibition of the FASN protein in mice resulted in reduced total body fat [55] and body weight [56], respectively. We have identified two SNPs in this gene in Mangalicas that result in a R443Q (SNP1) and a T1088I (SNP2) amino acid change. The amino acid in position 443 is part of the α-helix in the protein’s inter-domain linker. Since glutamine is more hydrophilic than arginine, the amino acid substitution may affect the relative position of the two functional domains by modulating the flexibility of the linker connecting them [57]. The amino acid in position 1,088 is part of the dehydratase domain of the FASN protein. This domain catalyses the conversion of β-hydroxyacyl-ACP to β-enoyl-ACP in the cyclic elongation of fatty acids [47]. T_1088_ is in close vicinity to the active site of the dehydratase domain containing an open-ended hydrophobic tunnel [57]. Predicting hydrophobicity of amino acids along the FASN polypeptide revealed that the substituting I_1088_ is strongly hydrophobic, while T_1088_ is hydrophilic (data not shown). It is possible, therefore, that in the FASN_T1088I_ protein the substrate-binding nature of the active site is altered, which may influence the dehydration step of the fatty acid cyclic elongation. This might be particularly important in Mangalicas, where no BB homozygotes were found. Thus the active site in the catalytic domain of their FASN protein is expected to be hydrophobic, although allele-specific expression of the *FASN* gene in heterozygotes might influence this.

It is known that feeding regimes influence fatty acid composition and meat’s marbling in Mangalicas [31, 58], similar to other pig breeds and farm animals. In lipid metabolism, the “Fat digestion and absorption” and “Bile secretion” pathways are involved in the metabolism of dietary fats. These two pathways are connected to the “Glycerolipid metabolism”, “Fatty acid metabolism” and “Fatty acid biosynthesis” pathways. Our study highlighted a number of genes in these metabolic pathways and in the PPAR signalling pathway (Figure 6). We have identified one gene, *MOGAT2* (ENSSSCG00000014861), with seven SNPs, of which four are present in Mangalicas, but not in other 56 sequenced pig individuals (see Results). The MOGAT2 protein catalyses the conversion of 1-acylglycerol obtained from dietary fat into diacylglycerol in the smooth endoplasmatic reticulum of the small intestinal epithelial cells, and thus participates in the production of chylomicron (“Fat digestion and absorption” pathway, KEGG:04975). Chylomicron affects the PPAR signalling pathway, which in turn regulates a number of lipid metabolic processes (Figure 6). It is possible, therefore, that polymorphisms that affect genes in this complex networks of pathways, which are also part of relevant QTLs, may be responsible for the differences in fattening, fat composition and any related phenotypes that were observed between breeds in response to different feeding regimes. For example, the *MOGAT2* gene was found to be part of the lipid concentration biological function, modulated in backfat [54].

## Conclusions

The discovery of genes behind agriculturally important traits is a difficult task in farm animals, in particular when the intermediate- or end-phenotypes are determined by QTLs. In this study, we described the genome sequencing and analysis of three Hungarian Mangalica individuals representing each of the three Mangalica breeds, which are local, fatty type pigs with a niche role in the pork market. After filtering, millions of SNPs were identified in each animal compared to the reference genome, and about 10% of them are novel compared to the porcine SNP entries of the dbSNP138 database. This finding highlights that sequencing genomes of individuals of rare/local breeds can provide large amounts of data identifying genomic variations relative to the reference genome of the same species. These variations can be the basis for gene discoveries. With special emphasis on pig fatness, by annotating and comparing exonic, non-synonymous Mangalica-specific SNPs to QTLs and pathways, we identified a number of candidate genes, which can serve for future genotyping, expression, structure-function, and biological network studies and in applications, such as molecular breeding and meat identification or tracing in both Mangalica and other breeds.

## Methods

### Genome sequencing

Pig blood samples were obtained from the MANGFOOD consortium’s Biobank at the Agricultural Biotechnology Center, Gödöllő, Hungary. Total DNA was extracted using the Duplicα® Prep Automatic Extraction System and the Duplicα® Blood DNA kit (EuroClone, Milan, Italy). DNA concentration was measured using the Quant-iT™ PicoGreen dsDNA® Assay (Life Technologies, Budapest, Hungary). Preparation of 500 bp fragment libraries and 2 × 100 bp Illumina paired-end genome sequencing was performed by Aros Applied Biotechnology (Aarhus, Denmark) as a custom service, using Illumina’s HiSeq2000 platform.

### Data analyses

The *Sus scrofa* reference genome sequence 10.2 was indexed using the “bwtsw” algorithm option of BWA 0.5.9rc1 [59] followed by mapping the short sequence reads to the indexed genome using the default settings and the paired-end method of the same software. The obtained BAM files were sorted and indexed for further analyses.

To detect small genetic variants (SNPs and INDELs), the SAMtools [60] and GATK (version: 2.3-9-ge5ebf34) [61] variant calling pipelines were employed. In SAMtools, base-calling was performed using the “mpileup” command and the “-E -D -S -u” parameters of SAMtools 0.1.18. The “view” command of BCFtools was used to call the variants using the “-bvcg” parameters. VCF files were then generated by the “vcfutils.pl” script using the “varFilter” option and SNPs and INDELs were extracted. Finally, SNPs, which had a Phred score higher than 30 (i.e. their base-calling accuracy is larger then 99.9%), and a high-quality read coverage of minimum three, were filtered using a custom script. INDELs were used in downstream analyses without filtering. For GATK, the dbSNP138 data were used as a training set. Other settings were used according to the GATK best practice online documentation. Results obtained by the two pipelines were compared using the BEDTools’ [62] “intersectBed” module for SNPs and using our custom script for INDELS; only concordant variations were processed further.

Copy number variations (CNVs) were detected as described by Paudel and coworkers [44] using the mrCaNaVar (version 0.51) software [63]. The window size was set to 1,000 bp. We selected windows where the copy number and the standard deviation were bigger than three and 0.7, respectively, for the three Mangalicas. After that step the regions were chained.

To determine novel variants in our sequence data, we compared the identified SNPs and INDELs with the dbSNP138 data using BEDTools [62] and annotated the detected genetic variants using ANNOVAR [64]. Following the ANNOVAR analysis, non-synonymous exonic SNPs, which were present only in Mangalicas, were determined by BEDTools’ “multiIntersectBed” module. Genes carrying these variants were identified using a custom script. Comparison of SNPs in the lipid metabolism genes amongst genome sequenced animals (this study and literature 44) were also performed using the “multiIntersectBed” module of BEDTools.

Gene ontology analysis was performed by the web-based software PANTHER [45]. For overrepresentation analyses, Biomart’s [65] enrichment analysis option with 0.05 cut off P-value was employed using the Sscrofa 10.2 reference genome as background. Random sets of genes was generated by a custom Python script. Fat-related pig QTLs and their positions were downloaded from the QTLdb (Release 19) database [46], and their extension was compared with the position of the SNPs of selected genes manually. Genes were annotated into pathways using the KEGG database.

Data from Ensembl were retrieved using BioMart [65]; Venn diagrams were generated using the software Venny [66]; clustering was performed using CIMminer [67] with Manhattan distance and complete linkage clustering settings.

### Genotyping

To genotype the two Mangalica-specific SNPs in the *FASN* gene, High Resolution Melting (HRM) analysis was performed with a Rotor-Gene Q 5plex HRM Platform using a saturating dye (EvaGreen) technology (Qiagen, Hilden, Germany). PCR reactions were performed in 25 μl reaction volumes using 60 ng total DNA as template and the Type-it HRM PCR kit (Qiagen, Hilden, Germany), according to the instruction of the manufacturer. The primers for *FASN* SNP1 and SNP2 were FASN1_F: 5′ CGCGATCTCGTTGAGCAT 3′, FASN1_R: 5′ GTGCAGACCCTGCTGGAG 3′ and FASN2_F: 5′ GGATAGGCTTGAGATGCTCTT 3′, FASN2_R 5′ GTGGTGGTGGACAGGAATCT 3′, respectively. Reactions were carried out with an initial denaturation step at 95°C for 5 min, followed by 35 cycles of 95°C for 15 sec, 60°C for 30 sec and 72°C for 10 sec and then HRM curves were generated by acquiring florescence data between 80 and 91°C. Individuals with homozygous and heterozygous genotypes were assigned according to their HRM curve determined by the Rotor-Gene software and visual inspection.

### Availability of supporting data

The data sets supporting the results of this article are included within the article and its additional files. Sequence data are deposited to the NCBI Sequence Read Archive under identifier SRP039012.

## Electronic supplementary material

Additional file 1: Figure S1: Distribution of insert length in paired-end sequencing. Figure showing the distribution of insert length and number of reads in four sequenced pig individuals. (PDF 22 KB)

Additional file 2: Figure S2: Comparison of SNPs and INDELs detected by SAMtools and GATK. The numbers in the overlapping areas represent the absolute number of concordant variants, while coloured numbers represent the percentage of unique variants. BM, Blond Mangalica; RM, Red Mangalica; SM, Swallow-belly Mangalica; D, Duroc. (PDF 4 KB)

Additional file 3: Table S1 and S2: Number of filtered SNPs. **Table S1.** The number of filtered SNPs in four sequenced pig individuals. **Table S2.** The number of filtered SNPs in the four animals that are present in the dbSNP 138 database. BM, Blond Mangalica; RM, Red Mangalica; SM, Swallow-belly Mangalica; D, Duroc. (PDF 34 KB)

Additional file 4: Table S3: Annotation of SNPs. Table showing the annotation of SNPs into categories and the ratio of heterozygous-homozygous SNPs in each category. (XLS 46 KB)

Additional file 5: Figure S3: Distribution of the size of INDELs. Figure showing the number of INDELs with respect to their size in four sequenced pig individuals. (PDF 23 KB)

Additional file 6: Table S4: Annotation of INDELs. Table showing the number and percentage of INDELs annotated into categories. (XLS 54 KB)

Additional file 7: Figure S4: Distribution of the size of exonic frame-shift INDELs. Figure showing the number of exonic frame-shift INDELs with respect to their size in four sequenced pig individuals. (PDF 23 KB)

Additional file 8: Table S5: Copy number variations in Mangalicas. Table showing CNVs found in Mangalica pigs, their overlap with a Duroc animal and published data [44], and the overrepresented GO category. (XLS 254 KB)

Additional file 9: Table S6: SNP variations in Mangalicas. Table showing SNPs identified in Mangalicas, which are not present in the dbSNP138 and the sequenced Duroc animal used in this study. Multiple SNPs of the same gene are shown in separate rows with gene ID in grey. Transcript and alternative transcript annotation of the SNP are shown in the “SNP Annotation” column. (XLS 464 KB)

Additional file 10: Table S7: Overrepresentation analysis of genes with exonic non-synonymous SNPs in Mangalicas. Represented GO categories of 1,389 genes, which carry SNPs identified in the three sequenced Mangalica individuals, and of two same size control pig gene sets. Overrepresentation can be considered statistically significant where the corrected P value is smaller than 5E-2. (PDF 5 KB)

Additional file 11: Table S8: Selected genes with Mangalica-specific exonic non-synonymous SNPs. Table showing the genes with Mangalica-specific SNPs that belong to the GO:0006629 lipid metabolic process category, their annotation to pathways and the QTL regions that overlap them. (XLS 95 KB)

Additional file 12: Table S9: Genes with Mangalica-specific SNPs in “Fatty acid percentage” QTLs. Table showing the name, Ensembl Gene ID, description and associated fatty acid percentage QTLs of genes with Mangalica-specific SNPs and belonging to the GO:0006629 lipid metabolic process category. (XLS 25 KB)

Additional file 13: Table S10: Occurrence of SNPs in 59 genome sequenced pig individuals. Table showing the presence (1) or absence (0) of those exonic, non-synonymous SNPs, which were identified in all three genome-sequenced Mangalicas, but not in the sequenced Duroc and the reference genome, and which are lipid metabolic genes. The genome sequenced individuals [9] are shown by their identification number and breed/species name. (XLS 148 KB)

Additional file 14: Figure S5: Heat-map of the frequency of 82 SNPs in genome sequenced pigs. Figure showing the clustering of 82 SNPs by their frequency in pig breeds/species. Four distinct clusters can be observed consisting of Mangalicas, European pigs/wild boars, Duroc of different origin and non-European pigs/wild boars/related species. The order of SPNs from top to bottom corresponds to those in Table S10 (Additional file 13). (PDF 209 KB)

Additional file 15: Figure S6: SNP frequencies in the *FASN* gene. Figure showing the frequencies of two SNPs in 59 genome sequenced pigs, including the four in this study and 55 published by Groenen et al. [9]. The number of individuals is shown with brackets after each name. *Sus*, three species, *Sus cebifrons*, *Sus celebensis* and *Sus verrucosus* from the genus; Other, Bearded pig and warthog; WE, Western European; Nature total, overall frequency of the published [9] 55 individuals. (PDF 21 KB)

## References

[1] Fadiel A, Anidi I, Eichenbaum KD (2005). Farm animal genomics and informatics: an update. Nucleic Acids Res.

[2] Rothschild MF, Plastow GS (2008). Impact of genomics on animal agriculture and opportunities for animal health. Trends Biotechnol.

[3] International Chicken Genome Sequencing Consortium (2004). Sequence and comparative analysis of the chicken genome provide unique perspectives on vertebrate evolution. Nature.

[4] Dalloul RA, Long JA, Zimin AV, Aslam L, Beal K, Blomberg LA, Bouffard P, Burt DW, Crasta O, Crooijmans RPMA, Cooper K, Coulombe RA, De S, Delany ME, Dodgson JB, Dong JJ, Evans C, Frederickson KM, Flicek P, Florea L, Folkerts O, Groenen MAM, Harkins TT, Herrero J, Hoffmann S, Megens HJ, Jiang A, de Jong P, Kaiser P, Kim H (2010). Multi-platform next-generation sequencing of the domestic turkey (*Meleagris gallopavo*): Genome assembly and analysis. PLoS Biol.

[5] Elsik CG, Tellam RL, Worley KC, The Bovine Genome Sequencing and Analysis Consortium (2009). The genome sequence of taurine cattle: A window to ruminant biology and evolution. Science.

[6] Wade CM, Giulotto E, Sigurdsson S, Zoli M, Gnerre S, Imsland F, Lear TL, Adelson DL, Bailey E, Bellone RR, Blocker H, Distl O, Edgar RC, Garber M, Leeb T, Mauceli E, MacLeod JN, Penedo MC, Raison JM, Sharpe T, Vogel J, Andersson L, Antczak DF, Biagi T, Binns MM, Chowdhary BP, Coleman SJ, Della Valle G, Fryc S, Guerin G (2009). Genome sequence, comparative analysis, and population genetics of the domestic horse. Science.

[7] Archibald AL, Cockett NE, Dalrymple BP, Faraut T, Kijas JW, Maddox JF, McEwan JC, Hutton Oddy V, Raadsma HW, Wade C, Wang J, Wang W, Xun X, The International Sheep Genomics Consortium (2010). The sheep genome reference sequence: a work in progress. Anim Genet.

[8] Dong Y, Xie M, Jiang Y, Xiao N, Du X, Zhang W, Tosser-Klopp G, Wang J, Yang S, Liang J, Chen W, Chen J, Zeng P, Hou Y, Bian C, Pan S, Li Y, Liu X, Wang W, Servin B, Sayre B, Zhu B, Sweeney D, Moore R, Nie W, Shen Y, Zhao R, Zhang G, Li J, Faraut T (2013). Sequencing and automated whole-genome optical mapping of the genome of a domestic goat (*Capra hircus*). Nature Biotechnol.

[9] Groenen MAM, Archibald AL, Uenishi H, Tuggle CK, Takeuchi Y, Rothschild MF, Rogel-Gaillard C, Park C, Milan D, Megens HJ, Li S, Larkin DM, Kim H, Frantz LAF, Caccamo M, Ahn H, Aken BL, Anselmo A, Anthon C, Auvil L, Badaoui B, Beattie CW, Bendixen C, Berman D, Blecha F, Blomberg J, Bolund L, Bosse M, Botti S, Bujie Z (2012). Analyses of pig genomes provide insight into porcine demography and evolution. Nature.

[10] Canavez FC, Luche DD, Stothard P, Leite KRM, Sousa-Canavez JM, Plastow G, Meidanis J, Souza MA, Feijao P, Moore SS, Camara-Lopes LH (2012). Genome sequence and assembly of *Bos indicus*. J Hered.

[11] Qiu Q, Zhang G, Ma T, Qian W, Wang J, Ye Z, Cao C, Hu Q, Kim J, Larkin DM, Auvil L, Capitanu B, Ma J, Lewin HA, Qian X, Lang Y, Zhou R, Wang L, Wang K, Xia J, Liao S, Pan S, Lu X, Hou H, Wang Y, Zang X, Yin Y, Ma H, Zhang J, Wang Z (2012). The yak genome and adaptation to life at high altitude. Nat Genet.

[12] (2012). USDA Foreign Agricultural Service. Livestock and poultry: world markets and trade.

[13] Chen K, Baxter T, Muir WM, Groenen MA, Schook LB (2007). Genetic resources, genome mapping and evolutionary genomics of the pig (*Sus scrofa*). Int J Biol Sci.

[14] Molnár J, Tóth G, Stéger V, Zsolnai A, Jánosi A, Mohr A, Szántó-Egész R, Tóth P, Micsinai A, Rátky J, Marincs F (2013). Mitochondrial D-loop analysis reveals low diversity in Mangalica pigs and their relationship to historical specimens. J Anim Breed Genet.

[15] Wernersson R, Schierup MH, Jørgensen FG, Gorodkin J, Panitz F, Stærfeldt HH, Christensen OF, Mailund T, Hornshøj H, Klein A, Wang J, Liu B, Hu S, Dong W, Li W, Wong GKS, Yu J, Wang J, Bendixen C, Fredholm M, Brunak S, Yang H, Bolund L (2005). Pigs in sequence space: A 0.66X coverage pig genome survey based on shotgun sequencing. BMC Genomics.

[16] Schook LB, Beever JE, Rogers J, Humphray S, Archibald A, Chardon P, Milan D, Rohrer G, Eversole K (2005). Swine Genome Sequencing Consortium (SGSC): a strategic roadmap for sequencing the pig genome. Comp Funct Genom.

[17] Archibald AL, Bolund L, Churcher C, Fredholm M, Groenen MAM, Harlizius B, Lee KT, Milan D, Rogers J, Rothschild MF, Uenishi H, Wang J, Schook LB, The Swine Genome Sequencing Consortium (2010). Pig genome sequence - analysis and publication strategy. BMC Genomics.

[18] Amaral AJ, Megens HJ, Kerstens HHD, Heuven HCM, Dibbits B, Crooijmans RPMA, den Dunnen JT, Groenen MAM (2009). Application of massive parallel sequencing to whole genome SNP discovery in the porcine genome. BMC Genomics.

[19] Kerstens HHD, Kollers S, Kommadath A, del Rosario M, Dibbits B, Kinders SM, Crooijmans RP, Groenen MAM (2009). Mining for single nucleotide polymorphisms in pig genome sequence data. BMC Genomics.

[20] Ramos AM, Crooijmans RPMA, Affara NA, Amaral AJ, Archibald AL, Beever JE, Bendixen C, Churcher C, Clark R, Dehais P, Hansen MS, Hedegaard J, Hu ZL, Kerstens HH, Law AS, Megens HJ, Milan D, Nonneman DJ, Rohrer GA, Rothschild MF, Smith TPL, Schnabel RD, Van Tassell CP, Taylor JF, Wiedmann RT, Schook LB, Groenen MAM (2009). Design of a high density SNP genotyping assay in the pig using SNPs identified and characterized by next generation sequencing technology. PLoS One.

[21] Zhang W, Wu W, Lin W, Zhou P, Dai L, Zhang Y, Huang J, Zhang D (2010). Deciphering heterogeneity in pig genome assembly Sscrofa9 by isochore and isochore-like region analyses. PLoS One.

[22] Damon M, Wyszynska-Koko J, Vincent A, Hérault F, Lebret B (2012). Comparison of muscle transcriptome between pigs with divergent meat quality phenotypes identifies genes related to muscle metabolism and structure. PLoS One.

[23] Zhao S, Hulsegge B, Harders FL, Bossers R, Keuning E, Hoekman AJW, Hoving-Bolink R, te Pas MFW (2013). Functional analysis of inter-individual transcriptome differential expression in pig longissimus muscle. J Anim Breed Genet.

[24] Dreher F, Kamburov A, Herwig R (2012). Construction of a pig physical interactome using sequence homology and a comprehensive reference human interactome. Evol Bioinform.

[25] Amaral AJ, Ferretti L, Megens H-J, Crooijmans RPMA, Nie H, Ramos-Onsins SE, Perez-Enciso M, Schook LB, Groenen MAM (2011). Genome-wide footprints of pig domestication and selection revealed through massive parallel sequencing of pooled DNA. PLoS One.

[26] *Companion articles for the publication of the swine genome*. [http://www.biomedcentral.com/series/swine/]

[27] Bosse M, Megens HJ, Madsen O, Paudel Y, Frantz LAF, Schook LB, Crooijmans RPMA, Groenen MAM (2012). Regions of homozygosity in the porcine genome: consequence of demography and the recombination landscape. PLoS Genet.

[28] Esteve-Codina A, Paudel Y, Ferretti L, Raineri E, Megens HJ, Silió L, Rodríguez MC, Groenen MAM, Ramos-Onsins SE, Pérez-Enciso M (2013). Dissecting structural and nucleotide genome-wide variation in inbred Iberian pigs. BMC Genomics.

[29] Fang X, Mu Y, Huang Z, Li Y, Han L, Zhang Y, Feng Y, Chen Y, Jiang X, Zhao W, Sun X, Xiong Z, Yang L, Liu H, Fan D, Mao L, Ren L, Liu C, Wang J, Li K, Wang G, Yang S, Lai L, Zhang G, Li Y, Wang J, Bolund L, Yang H, Wang J, Feng S, Li S, Du Y (2012). The sequence and analysis of a Chinese pig genome. GigaScience.

[30] Hall SJG, Bradley DG (1995). Conserving livestock breed biodiversity. Trends Ecol Evol.

[31] Szabó A, Viski A, Egyházi Z, Házas Z, Horn P, Romvári R (2010). Comparison of Mangalica and Hungarian Large White pigs at identical bodyweight: 1 Backfat histology. Arch Tierzucht.

[32] Switonski M, Stachowiak M, Cieslak J, Bartz M, Grzes M (2010). Genetics of fat tissue accumulation in pigs: a comparative approach. J Appl Genet.

[33] Zsolnai A, Radnóczy L, Fésüs L, Anton I (2006). Do Mangalica pigs of different colours really belong to different breeds?. Arch Tierzucht.

[34] Egerszegi I, Schneider F, Rátky J, Soós F, Solti L, Manabe N, Brüssow KP (2003). Comparison of luteinizing hormone and steroid hormone secretion during the peri- and post-ovulatory periods in Mangalica and Landrace gilts. J Reprod Develop.

[35] Rátky J, Brüssow KP, Egerszegi I, Torner H, Schneider F, Solti L, Manabe N (2005). Comparison of follicular and oocyte development and reproductive hormone secretion during the ovulatory period in Hungarian native breed, Mangalica, and Landrace gilts. J Reprod Develop.

[36] Egerszegi I, Hazeleger W, Rátky J, Sarlós P, Kemp B, Bouwman E, Solti L, Brüssow KP (2007). Superovulatory ovarian response in Mangalica gilts is not influenced by feeding level. Reprod Domest Anim.

[37] Brüssow KP, Schneider F, Tuchscherer A, Egerszegi I, Rátky J (2008). Comparison of luteinizing hormone, leptin and progesterone levels in the systemic circulation (Vena jugularis) and near the ovarian circulation (Vena cava caudalis) during the oestrous cycle in Mangalica and Landrace gilts. J Reprod Develop.

[38] Sarlós P, Egerszegi I, Nagy S, Fébel H, Rátky J (2011). Reproductive function of Hungarian Mangalica boars: effect of seasons. Acta Vet Hung.

[39] Drögemüller C, Giese A, Martins-Wess F, Wiedemann S, Andersson L, Brenig B, Fries R, Leeb T (2006). The mutation causing the black-and-tan pigmentation phenotype of Mangalitza pigs maps to the porcine ASIP locus but does not affect its coding sequence. Mamm Genome.

[40] Marincs F, Molnár J, Tóth G, Stéger V, Barta E (2013). Introgression and isolation contributed to the development of Hungarian Mangalica pigs from a particular European ancient bloodline. Genet Sel Evol.

[41] Ursing BM, Arnason U (1998). The complete mitochondrial DNA sequence of the pig (Sus scrofa). J Mol Evol.

[42] Pelak K, Shianna KV, Ge D, Maia JM, Zhu M, Smith JP, Cirulli ET, Fellay J, Dickson SP, Gumbs CE, Heinzen EL, Need AC, Ruzzo EK, Singh A, Campbell CR, Hong LK, Lornsen KA, McKenzie AM, Sobreira NLM, Hoover-Fong JE, Milner JD, Ottman R, Haynes BF, Goedert JJ, Goldstein DB (2010). The Characterization of Twenty Sequenced Human Genomes. PLoS Genet.

[43] Ng PC, Levy S, Huang J, Stockwell TB, Walenz BP, Li K, Axelrod N, Busam DA, Strausberg RL, Venter JC (2008). Genetic Variation in an Individual Human Exome. PLoS Genet.

[44] Paudel Y, Madsen O, Megens HJ, Frantz LAF, Bosse M, Bastiaansen JWM, Crooijmans RPMA, Groenen MAM (2013). Evolutionary dynamics of copy number variation in pig genomes in the context of adaptation and domestication. BMC Genomics.

[45] Mi H, Muruganujan A, Thomas PD (2013). PANTHER in 2013: modeling the evolution of gene function, and other gene attributes, in the context of phylogenetic trees. Nucleic Acid Res.

[46] Hu ZL, Park CA, Wu XL, Reecy JM (2013). Animal QTLdb: an improved database tool for livestock animal QTL/association data dissemination in the post-genome era. Nucleic Acids Res.

[47] Maier T, Jenni S, Ban N (2006). Architecture of Mammalian Fatty Acid Synthase at 4.5 Å Resolution. Science.

[48] Gallardo D, Amills M, Quintanilla R, Pena RN (2013). Mapping and tissue mRNA expression analysis of the pig solute carrier 27A (SLC27A) multigene family. Gene.

[49] Mandard S, Patsouris D (2013). Nuclear Control of the Inflammatory Response in Mammals by Peroxisome Proliferator-Activated Receptors. PPAR Res.

[50] Muñoz G, Alves E, Fernández A, Óvilo C, Barragán C, Estellé J, Quintanilla R, Folch JM, Silió L, Rodríguez MC, Fernández AI (2007). QTL detection on porcine chromosome 12 for fatty-acid composition and association analyses of the fatty acid synthase, gastric inhibitory polypeptide and acetyl-coenzyme A carboxylase alpha genes. Anim Genet.

[51] Cánovas A, Quintanilla R, Amills M, Pena RN (2010). Muscle transcriptomic profiles in pigs with divergent phenotypes for fatness traits. BMC Genomics.

[52] Stachowiak M, Nowacka-Woszuk J, Szydlowski M, Switonski M (2013). The ACACA and SREBF1 genes are promising markers for pig carcass and performance traits, but not for fatty acid content in the longissimus dorsi muscle and adipose tissue. Meat Sci.

[53] Estellé J, Fernández AI, Pérez-Enciso M, Fernández A, Rodríguez C, Sánchez A, Noguera JL, Folch JM (2009). A non-synonymous mutation in a conserved site of the *MTTP* gene is strongly associated with protein activity and fatty acid profile in pigs. Anim Genet.

[54] Corominas J, Ramayo-Caldas Y, Puig-Oliveras A, Estellé J, Castelló A, Alves E, Pena RN, Ballester M, Folch JM (2013). Analysis of porcine adipose tissue transcriptome reveals differences in de novo fatty acid synthesis in pigs with divergent muscle fatty acid composition. BMC Genomics.

[55] Chakravarthy MV, Pan Z, Zhu Y, Tordjman K, Schneider JG, Coleman T, Turk J, Semenkovich CF (2005). 'New' hepatic fat activates PPARalpha to maintain glucose, lipid, and cholesterol homeostasis. Cell Metab.

[56] Loftus TM, Jaworsky DE, Frehywot GL, Townsend CA, Ronnett GV, Lane MD, Kuhajda FP (2000). Reduced food intake and body weight in mice treated with fatty acid synthase inhibitors. Science.

[57] Maier T, Leibundgut M, Ban N (2008). The Crystal Structure of a Mammalian Fatty Acid Synthase. Science.

[58] Szabó A, Horn P, Romvári R, Házas Z, Fébel H (2010). Comparison of Mangalica and Hungarian Large White pigs at identical bodyweight: 2. Fatty acid regiodistribution analysis of the triacylglycerols. Arch Tierzucht.

[59] Li H, Durbin R (2010). Fast and accurate long-read alignment with Burrows-Wheeler transformation. Bioinformatics.

[60] Li H, Handsaker B, Wysoker A, Fennell T, Ruan J, Homer N, Marth G, Abecasis G, Durbin R, The 1000 Genome Project Data Processing Subgroup (2009). The Sequence Alignment/Map format and SAMtools. Bioinformatics.

[61] DePristo MA, Banks E, Poplin RV, Garimella KV, Maguire JR, Hartl C, Philippakis AA, del Angel G, Rivas MA, Hanna M, McKenna A, Fennell TJ, Kernytsky AM, Sivachenko AY, Cibulskis K, Gabriel SB, Altshuler D, Daly MJ (2011). A framework for variation discovery and genotyping using next-generation DNA sequencing data. Nat Genet.

[62] Quinlan AR, Hall IM (2010). BEDTools: a flexible suite of utilities for comparing genomic features. Bioinformatics.

[63] Alkan C, Kidd JM, Marques-Bonet T, Aksay G, Antonacci F, Hormozdiari F, Kitzman JO, Baker C, Malig M, Mutlu O, Sahinalp SC, Gibbs RA, Eichler EE (2009). Personalized copy number and segmental duplication maps using next-generation sequencing. Nat Genet.

[64] Wang K, Li M, Hakonarson H (2010). ANNOVAR: functional annotation of genetic variants from high-throughput sequencing data. Nucleic Acids Res.

[65] Kasprzyk A (2011). BioMart: driving a paradigm change in biological data management. Database.

[66] Oliveros JC (2007). VENNY: An interactive tool for comparing lists with Venn Diagrams.

[67] Weinstein JN, Myers TG, O’Connor PM, Friend SH, Fornace AJ, Kohn KW, Fojo T, Bates SE, Rubinstein LV, Anderson NL, Buolamwini JK, van Osdol WW, Monks AP, Scudiero DA, Sausville EA, Zaharevitz DW, Bunow B, Viswanadhan VN, Johnson GS, Wittes RE, Paull KD (1997). An information-intensive approach to the molecular pharmacology of cancer. Science.

