# Supplementary figures and images for: Genome sequencing and analysis of Mangalica, a fatty local pig of Hungary
Source: BMC Genomics. 2014 Sep 5;15(1):761. doi: 10.1186/1471-2164-15-761 (PMC4162939; doi:10.1186/1471-2164-15-761)

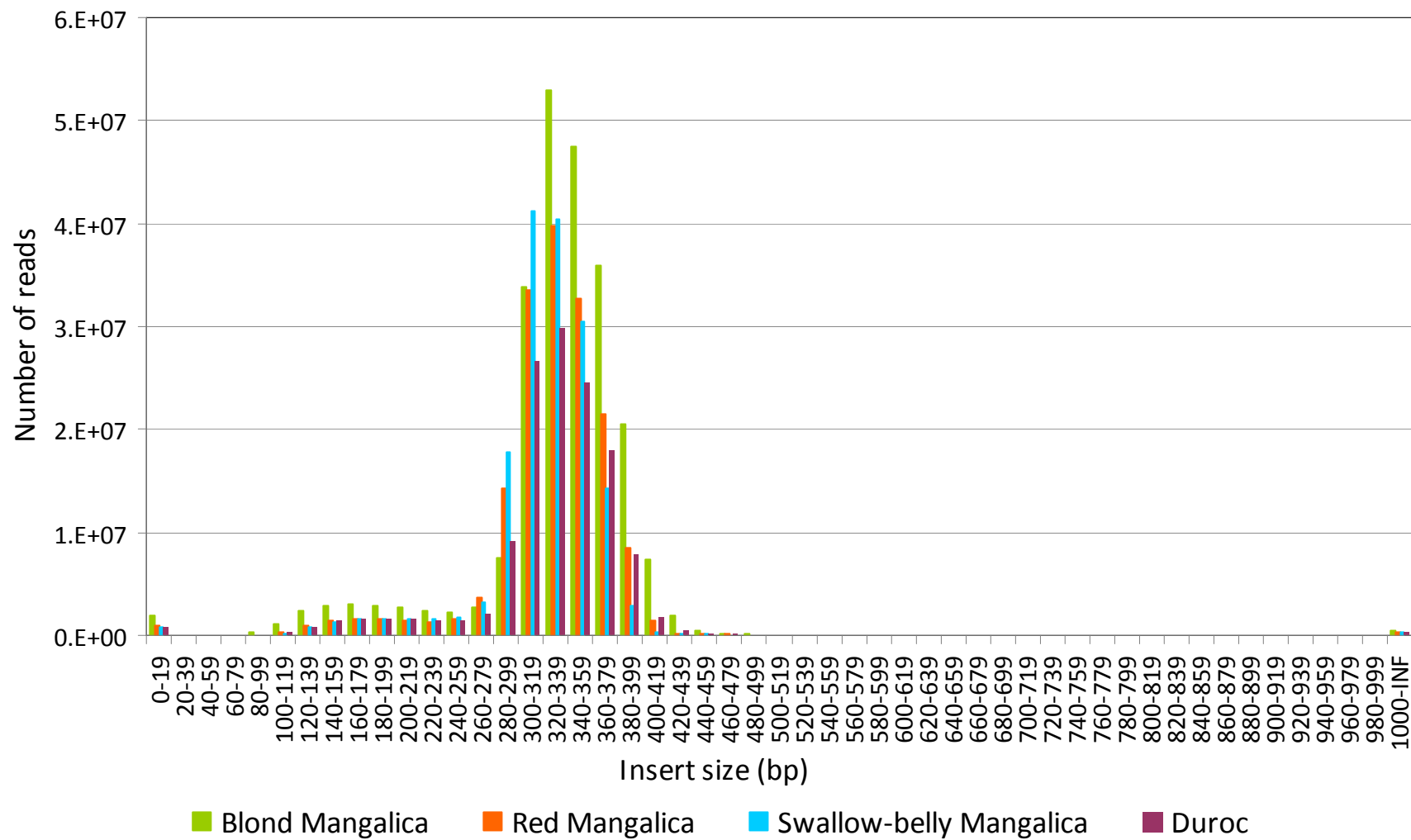

Supplement: Supplementary file 1 — Additional file 1: Figure S1: Distribution of insert length in paired-end sequencing. Figure showing the distribution of insert length and number of reads in four sequenced pig individuals. (PDF 22 KB) [file 12864_2013_6434_MOESM1_ESM.pdf]

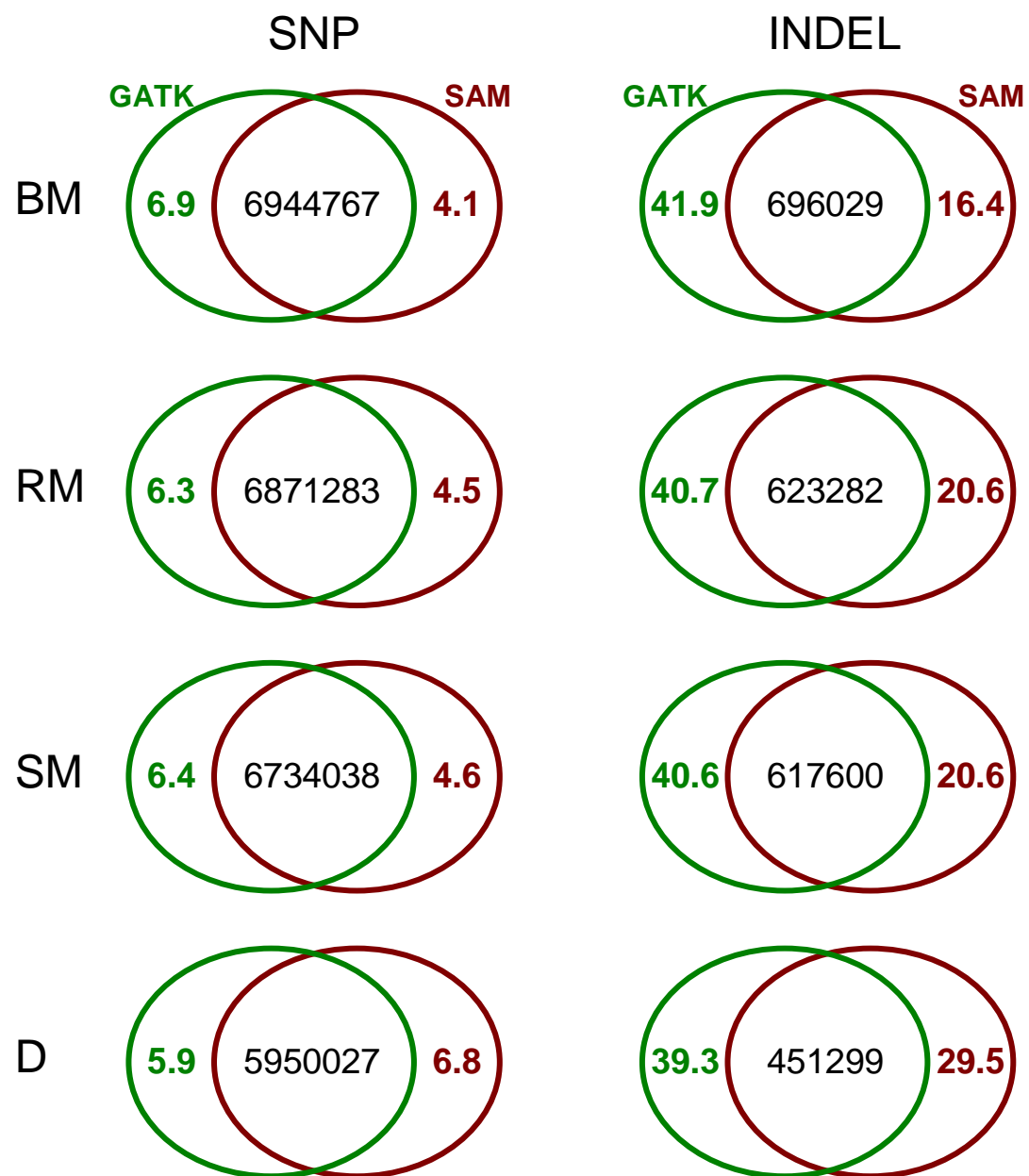

Supplement: Supplementary file 2 — Additional file 2: Figure S2: Comparison of SNPs and INDELs detected by SAMtools and GATK. The numbers in the overlapping areas represent the absolute number of concordant variants, while coloured numbers represent the percentage of unique variants. BM, Blond Mangalica; RM, Red Mangalica; SM, Swallow-belly Mangalica; D, Duroc. (PDF 4 KB) [file 12864_2013_6434_MOESM2_ESM.pdf]

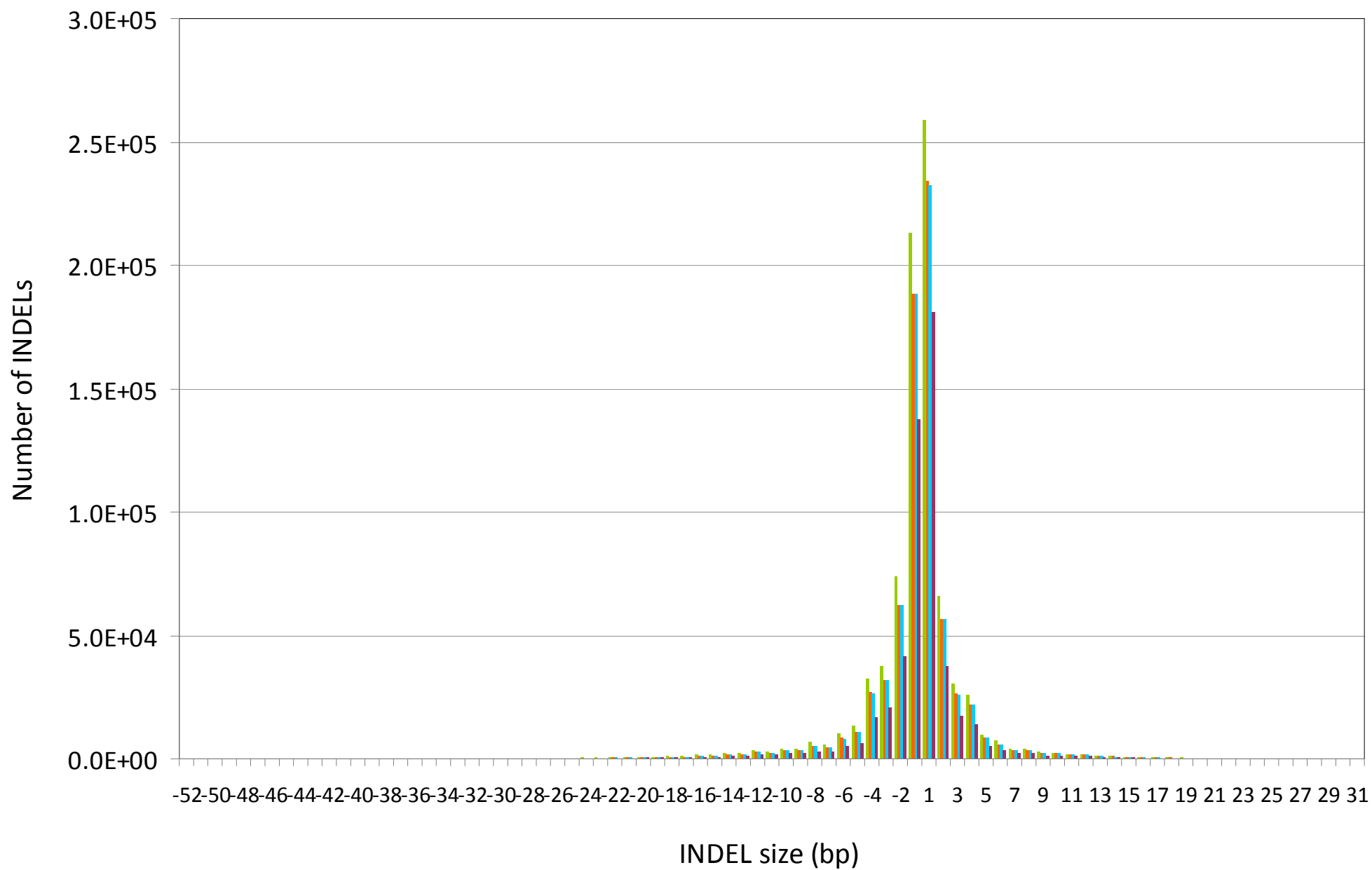

Supplement: Supplementary file 5 — Additional file 5: Figure S3: Distribution of the size of INDELs. Figure showing the number of INDELs with respect to their size in four sequenced pig individuals. (PDF 23 KB) [file 12864_2013_6434_MOESM5_ESM.pdf]

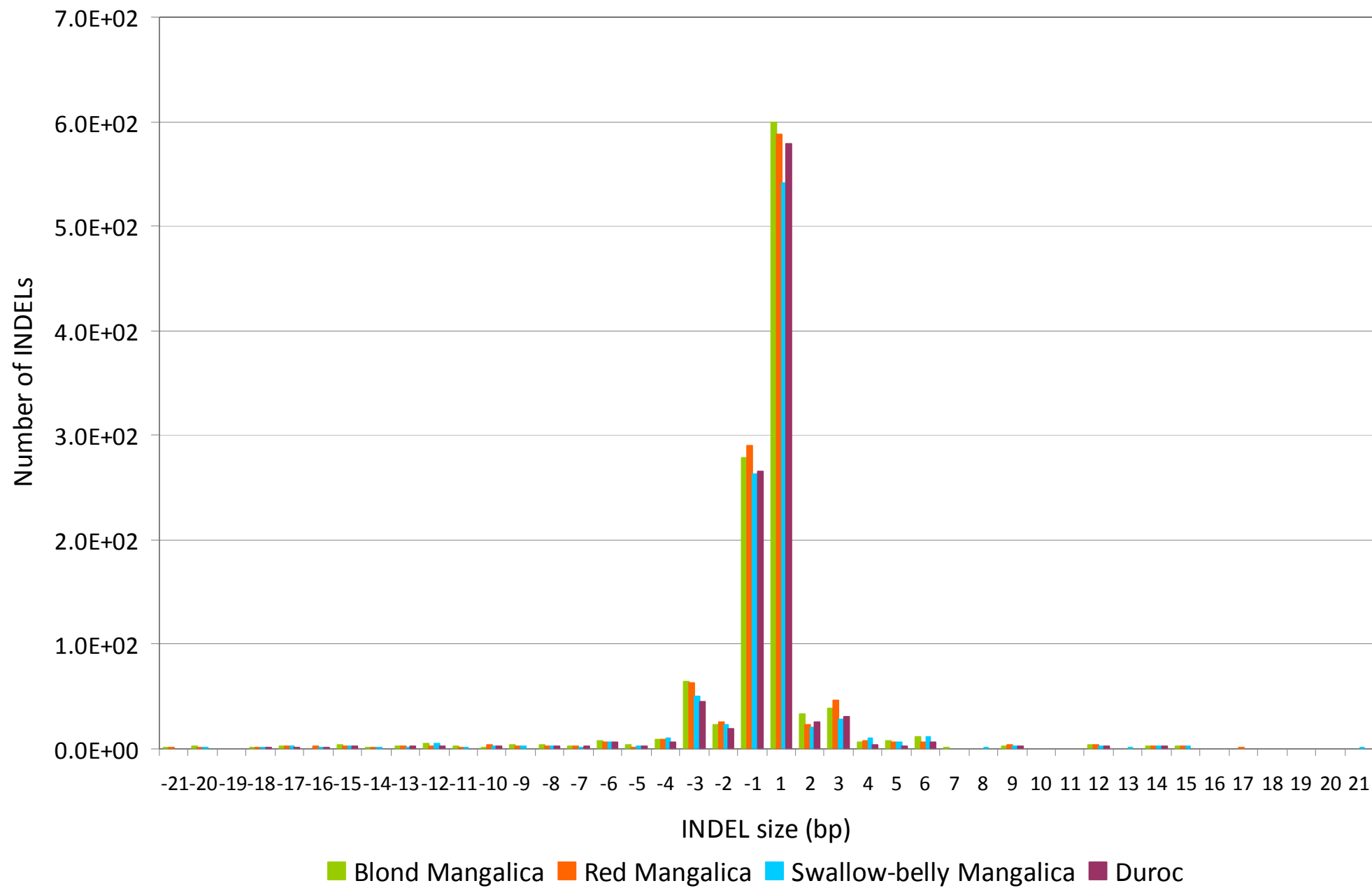

Supplement: Supplementary file 7 — Additional file 7: Figure S4: Distribution of the size of exonic frame-shift INDELs. Figure showing the number of exonic frame-shift INDELs with respect to their size in four sequenced pig individuals. (PDF 23 KB) [file 12864_2013_6434_MOESM7_ESM.pdf]

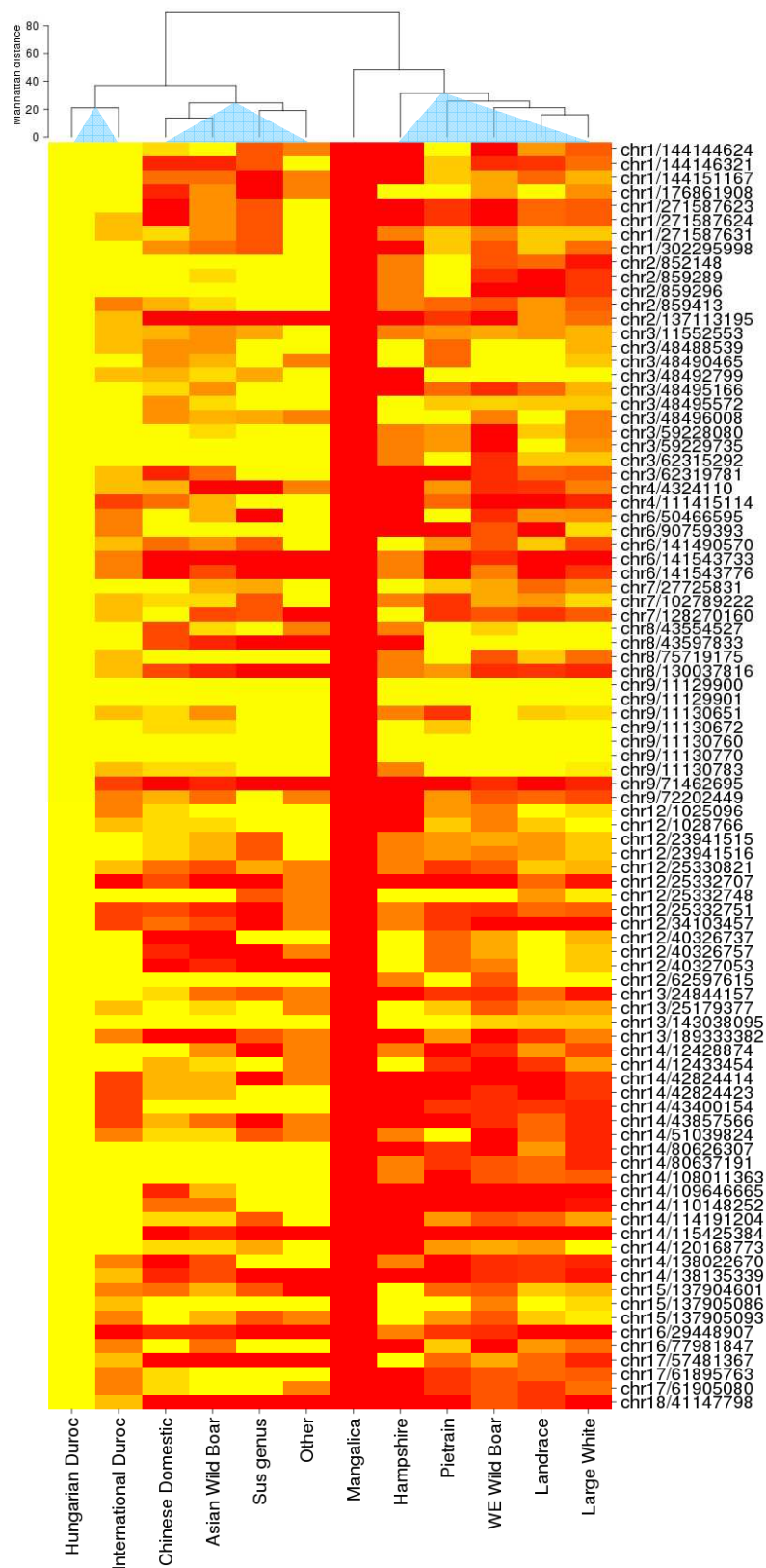

Supplement: Supplementary file 14 — Additional file 14: Figure S5: Heat-map of the frequency of 82 SNPs in genome sequenced pigs. Figure showing the clustering of 82 SNPs by their frequency in pig breeds/species. Four distinct clusters can be observed consisting of Mangalicas, European pigs/wild boars, Duroc of different origin and non-European pigs/wild boars/related species. The order of SPNs from top to bottom corresponds to those in Table S10 (Additional file 13). (PDF 209 KB) [file 12864_2013_6434_MOESM14_ESM.pdf]

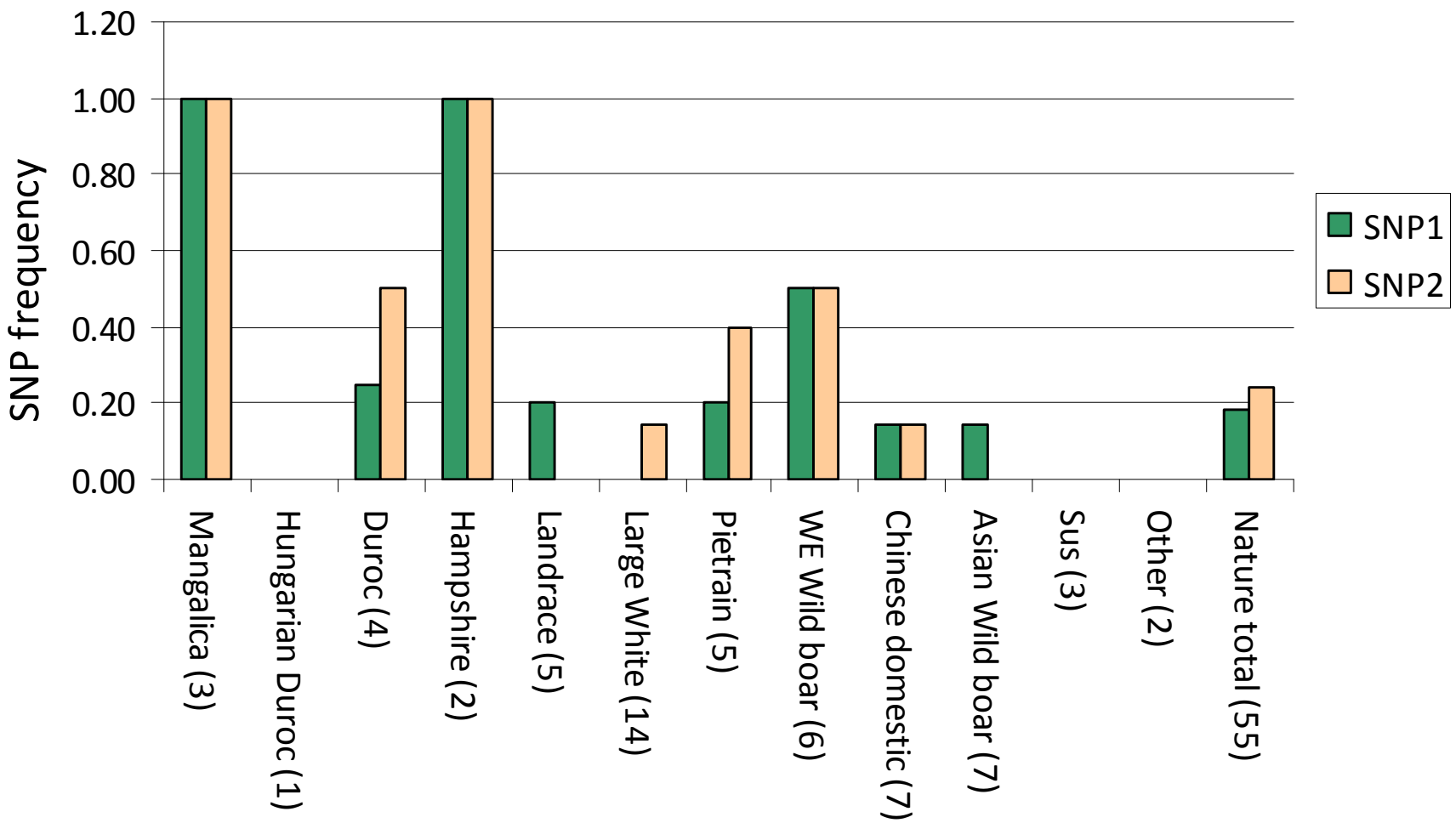

Supplement: Supplementary file 15 — Additional file 15: Figure S6: SNP frequencies in the FASN gene. Figure showing the frequencies of two SNPs in 59 genome sequenced pigs, including the four in this study and 55 published by Groenen et al. [9]. The number of individuals is shown with brackets after each name. Sus, three species, Sus cebifrons, Sus celebensis and Sus verrucosus from the genus; Other, Bearded pig and warthog; WE, Western European; Nature total, overall frequency of the published [9] 55 individuals. (PDF 21 KB) [file 12864_2013_6434_MOESM15_ESM.pdf]
