# Supplementary material for: Genome sequencing and analysis of Mangalica, a fatty local pig of Hungary
Source: BMC Genomics. 2014 Sep 5;15(1):761. doi: 10.1186/1471-2164-15-761 (PMC4162939; doi:10.1186/1471-2164-15-761)
Supplement: Supplementary file 3 — Additional file 3: Table S1 and S2: Number of filtered SNPs. Table S1. The number of filtered SNPs in four sequenced pig individuals. Table S2. The number of filtered SNPs in the four animals that are present in the dbSNP 138 database. BM, Blond Mangalica; RM, Red Mangalica; SM, Swallow-belly Mangalica; D, Duroc. (PDF 34 KB) [file 12864_2013_6434_MOESM3_ESM.pdf]

Table S1. The number of filtered SNPs identified in the sequenced individuals.

**Filtering**	**BM [a]**	**RM [a]**	**SM [a]**	**D [a]**
None (total number of SNPs)	6,944,767	6,871,283	6,734,038	5,950,027
Min. coverage = 3 high quality reads	6,323,065	6,486,448	6,386,803	5,657,769
Min. coverage = 3 high quality reads and Phred score ≥ 30	6,226,090	6,356,083	6,255,638	5,467,011

[a] BM, Blond Mangalica; RM, Red Mangalica; SM, Swallow-belly Mangalica, D, Duroc.

Table S2. The number of filtered SNPs in the dbSNP138 database.

**Versus dbSNP**	**BM [a]**	**RM [a]**	**SM [a]**	**D [a]**
Existing	5,679,568	5,623,387	5,508,731	4,848,800
Novel	546,522	732,696	746,907	618,211
Novel (%)	9.6	13.0	13.6	12.7

[a] BM, Blond Mangalica; RM, Red Mangalica; SM, Swallow-belly Mangalica, D, Duroc.

The sum of existing and novel SNPs in Table S2 for each animal equals to the coverage- and Phred score-filtered SNPs from Table S1.
