# Supplementary material for: Genome sequencing and analysis of Mangalica, a fatty local pig of Hungary
Source: BMC Genomics. 2014 Sep 5;15(1):761. doi: 10.1186/1471-2164-15-761 (PMC4162939; doi:10.1186/1471-2164-15-761)
Supplement: Supplementary file 10 — Additional file 10: Table S7: Overrepresentation analysis of genes with exonic non-synonymous SNPs in Mangalicas. Represented GO categories of 1,389 genes, which carry SNPs identified in the three sequenced Mangalica individuals, and of two same size control pig gene sets. Overrepresentation can be considered statistically significant where the corrected P value is smaller than 5E-2. (PDF 5 KB) [file 12864_2013_6434_MOESM10_ESM.pdf]

Mangalica gene-set

GO ID	Description	P value	Corrected P value
GO:0050877	neurological system process	6.3790E-22	9.1220E-20
GO:0005886	plasma membrane	6.5840E-18	9.4150E-16
GO:0004871	signal transducer activity	2.1053E-17	3.0105E-15
GO:0007165	signal transduction	8.0148E-10	1.1461E-07
GO:0005575	cellular component	1.2364E-04	1.7600E-02
GO:0005623	cell	6.2976E-04	9.0000E-02
GO:0005578	proteinaceous extracellular matrix	1.8659E-03	2.6680E-01
GO:0003674	molecular function	5.1215E-03	7.3230E-01
GO:0005730	nucleolus	5.3871E-03	7.7030E-01
GO:0005576	extracellular region	1.1732E-02	1.0000E+00
GO:0006629	lipid metabolic process	1.4711E-02	1.0000E+00
GO:0005856	cytoskeleton	2.3362E-02	1.0000E+00
GO:0005929	cilium	3.4435E-02	1.0000E+00
GO:0016798	hydrolase activity, acting on glycosyl bonds	3.6784E-02	1.0000E+00

Random gene-set I.

GO ID	Description	P value	Corrected P value
GO:0006520	cellular amino acid metabolic process	1.3935E-02	1.0000E+00
GO:0044403	symbiosis, encompassing mutualism through parasitism	3.2232E-02	1.0000E+00
GO:0006412	translation	3.5095E-02	1.0000E+00
GO:0030198	extracellular matrix organization	3.6090E-02	1.0000E+00
GO:0016874	ligase activity	4.0855E-02	1.0000E+00
GO:0005856	cytoskeleton	4.0974E-02	1.0000E+00

Random gene-set II.

GO ID	Description	P value	Corrected P value
GO:0006399	tRNA metabolic process	1.8675E-02	1.0000E+00
GO:0008135	translation factor activity, nucleic acid binding	4.0321E-02	1.0000E+00
GO:0001071	nucleic acid binding transcription factor activity	4.4022E-02	1.0000E+00
GO:0000003	reproduction	4.4084E-02	1.0000E+00
GO:0048646	anatomical structure formation involved in morphogenesis	4.4100E-02	1.0000E+00
GO:0016491	oxidoreductase activity	4.4204E-02	1.0000E+00
